# Temperature and solids retention time control microbial population dynamics and volatile fatty acid production in replicated anaerobic digesters

**DOI:** 10.1038/srep08496

**Published:** 2015-02-16

**Authors:** Inka Vanwonterghem, Paul D. Jensen, Korneel Rabaey, Gene W. Tyson

**Affiliations:** 1 Advanced Water Management Centre (AWMC), The University of Queensland, St Lucia, QLD 4072, Australia; 2 Australian Centre for Ecogenomics (ACE), School of Chemistry and Molecular Biosciences, The University of Queensland, St Lucia, QLD 4072, Australia; 3 Laboratory for Microbial Ecology and Technology (LabMET), Ghent University, Coupure Links 653, 9000 Ghent, Belgium

## Abstract

Anaerobic digestion is a widely used technology for waste stabilization and generation of biogas, and has recently emerged as a potentially important process for the production of high value volatile fatty acids (VFAs) and alcohols. Here, three reactors were seeded with inoculum from a stably performing methanogenic digester, and selective operating conditions (37°C and 55°C; 12 day and 4 day solids retention time) were applied to restrict methanogenesis while maintaining hydrolysis and fermentation. Replicated experiments performed at each set of operating conditions led to reproducible VFA production profiles which could be correlated with specific changes in microbial community composition. The mesophilic reactor at short solids retention time showed accumulation of propionate and acetate (42 ± 2% and 15 ± 6% of COD_hydrolyzed_, respectively), and dominance of *Fibrobacter* and *Bacteroidales*. Acetate accumulation (>50% of COD_hydrolyzed_) was also observed in the thermophilic reactors, which were dominated by *Clostridium*. Under all tested conditions, there was a shift from acetoclastic to hydrogenotrophic methanogenesis, and a reduction in methane production by >50% of COD_hydrolyzed_. Our results demonstrate that shortening the SRT and increasing the temperature are effective strategies for driving microbial communities towards controlled production of high levels of specific volatile fatty acids.

Anaerobic digestion (AD) is the conversion of organic matter to methane-rich biogas through a series of interlinked microbial processes: hydrolysis, fermentation, acetogenesis and methanogenesis. AD is a globally important technology in the fields of bio-energy and organic waste management, and has been successfully applied for sludge stabilization in wastewater treatment plants and for the production of biogas from energy crops and waste streams from industry and agriculture^1^,^2^,^3^,^4^,^5^. To date, most AD research has focused on improving process kinetics to maximize the organic loading rate, methane yields and energy recovery; and improving process stability by minimizing accumulation of intermediary products.

The carboxylate platform, previously known as the MixAlco process^6^, has reemerged as a biorefinery platform which uses microbial communities to convert complex substrates into valuable short-chain carboxylates, including volatile fatty acids (VFAs) such as acetate, propionate, butyrate and caproate^7^. The production of short-chain fatty acids from cellulosic feedstocks has important biotechnological potential as these carboxylates can be used as substrates for production of biofuels and bioplastics, or in other bioprocesses^8^. Anaerobic digestion has been included within this platform as VFAs are key intermediates in the production of methane^7^. Given the lower market value of methane relative to carboxylates^7^, suppression of biogas production within AD through restriction of methanogenesis, and stimulation of VFA production through enhanced hydrolysis and fermentation would be desirable.

Operating conditions including temperature, solids retention time (SRT) and substrate composition strongly influence AD process parameters such as stability and product formation. Several digester configurations have been developed, including multi-stage AD and temperature phased AD (TPAD), which exploit differences in substrate affinity, optimal growth conditions and maximum growth rates between bacterial and archaeal populations involved in AD, and promote different metabolic steps in separate reactor vessels^3^,^9^,^10^. Thermophilic AD (50–70°C) has several advantages over mesophilic AD (30–40°C), including increased hydrolysis rate, higher biogas production and improved pathogens destruction^3^,^11^,^12^. The operating temperature also regulates fermentation leading to increased production rates and changes in the composition of soluble products such as VFAs and alcohols^13^. As a result, there may be an imbalance between functional guilds at higher temperatures, leading to VFA accumulation and possible inhibition of methanogenesis^5^,^11^,^14^.

SRT, the average time solids (particulate substrates and microorganisms) are retained in the process vessel, is also an important parameter in AD operation as it determines the time available for substrate degradation and microbial growth. When the SRT is less than the growth rate of key members of the microbial community, biomass washout will occur leading to process failure. Methanogens are generally accepted as the slowest growing populations within AD, and under mesophilic conditions are strongly impacted by reducing the SRT below 6 days^3^,^14^. Therefore, shortening the SRT is recognized as an effective strategy for restricting methanogenesis while maintaining hydrolysis and fermentation, leading to accumulation of VFAs. Traditionally, VFA accumulation has been regarded as a sign of process failure in anaerobic digestion, however it is a desired outcome for fermentation processes within the carboxylate platform.

The selective pressure imposed by bioreactor operating conditions has a significant influence on microbial communities^15^,^16^,^17^,^18^,^19^,^20^. In order to explain the factors driving community assembly and dynamics, several theories have been adopted from macroecology. According to the traditional niche-based theory, there is a dominant influence of deterministic processes and the relationship between taxon traits and the environment. Neutral theory on the other hand rejects competition between populations and only considers stochastic processes, including birth, death, colonization and dispersal^21^,^22^,^23^,^24^. Recent studies using replicated experiments have reported that deterministic rather than stochastic processes play a dominant role in microbial community dynamics in controlled engineered systems^16^,^17^,^18^,^19^, highlighting the potential for stable and controlled product formation by microbial communities under specific conditions.

In this study, a replicated experiment was performed with three reactors set up at different temperature and SRT conditions, and seeded with inoculum from a stably performing AD enriched for cellulose hydrolysis. The sets of operating conditions were chosen to selectively drive the microbial communities in each reactor towards enhanced production of specific VFAs. The microbial community dynamics, i.e. changes in community composition under the new operating conditions, were examined to determine the level of inhibition and/or washout of methanogens and concurrent changes in bacterial populations. Community composition changes were correlated with operating conditions and VFA profiles. The findings from this study demonstrate that through the application of selective operating conditions, AD microbial communities can be reproducibly driven toward simultaneous stable production of high levels of specific VFAs with lower levels of methane from a cellulosic feedstock.

## Results

### Anaerobic digester performance at reduced SRT and/or increased temperature

A cellulose degrading parent reactor operated for 250 days under mesophilic conditions (37°C) and at a 12 day SRT (M12) was used to seed three experimental reactors (M4, T12 and T4) operated at different temperatures (37°C and 55°C) and SRTs (4 days and 12 days). Temperature and SRT were modified to drive AD microbial communities towards enhanced VFA accumulation by restricting methanogenesis through inhibition or biomass washout, respectively, while maintaining high rates of hydrolysis and fermentation. The experimental reactors were set up and run with identical operating conditions in two independent experiments (Exp1 and Exp2; Exp1 for 34 days and Exp2 for 90 days) to assess if changes in temperature and SRT have a reproducible effect on microbial community dynamics and reactor performance.

#### Mesophilic – 12 day SRT parent reactor (M12)

**Figure 1:**
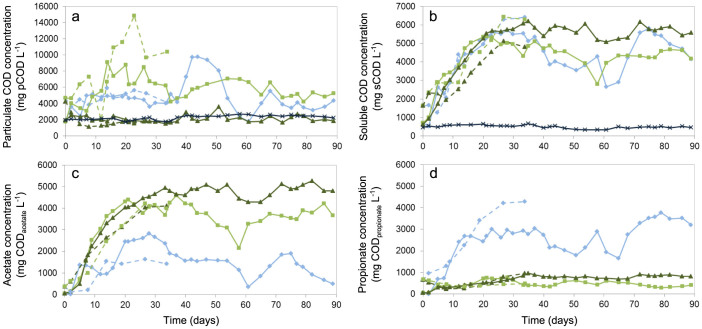
Reactor performance parameters over time for the parent (M12: ×dark blue) and experimental reactors (M4: 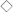 light blue; T12: Δ dark green; T4: 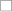 light green) during both experiments (Exp1: dashed line; Exp2: full line). The following concentrations were measured: (a) Particulate COD (pCOD); (b) Soluble COD (sCOD); (c) Acetate; (d) Propionate. Acetate and propionate concentrations are not shown for M12 as they were below 50 mg COD_VFA_ L^−1^. Reducing the SRT and/or increasing the temperature resulted in VFA accumulation, and distinct differences in VFA products could be observed between the mesophilic and thermophilic conditions.

The M12 reactor demonstrated stable performance for 250 days prior to Exp1. The residual cellulose concentration, represented by the particulate COD (pCOD), was 2.2 ± 0.3 g pCOD L^−1^ (Table 1 and Figure 1a), corresponding to a cellulose hydrolysis efficiency of 80 ± 5% (Supplementary Figure S1). The residual VFA concentration was 40 ± 16 mg COD_VFA_ L^−1^, indicating highly efficient conversion of VFAs to methane at an average methane production rate of 680 ± 150 mg COD_CH4_ L^−1^ d^−1^ (Table 1 and Supplementary Figure S2). The CH_4_ concentration in the biogas was 52 ± 3 vol%, CO_2_ concentration was 42 ± 5 vol% and H_2_ was not detected (<0.01 vol%). The performance of the parent reactor remained stable during both experiments, indicative of a well-functioning methanogenic digester.

#### Mesophilic – 4 day SRT reactor (M4)

Shortening the SRT resulted in a rapid increase in the residual cellulose concentration to maximum 4.7 g pCOD L^−1^ within 10 days in both experiments (Figure 1a). The pCOD concentration remained stable during Exp1 and the average cellulose hydrolysis efficiency was 61 ± 6%. During Exp2, reactor performance was less stable and fluctuations in pCOD concentrations were observed between Days 37 and 68 (Figure 1a). Between Days 15 and 37, the average cellulose hydrolysis efficiency was 63 ± 5%, comparable to Exp1, and increased over time to 69 ± 3% after Day 68 (Supplementary Figure S1). Significant accumulation of VFAs was observed at a 4 day SRT in contrast to the parent reactor (M12) with a 12 day SRT. VFA concentrations increased to a maximum of 6.4 g COD_VFA_ L^−1^ during Exp1 (Figure 1b). Soluble COD and VFA concentrations fluctuated during Exp2 between Days 37 and 68, before reaching an average of 5.0 ± 0.6 g COD_VFA_ L^−1^ after Day 68 (Table 1 and Figure 1b). In both experiments, the dominant VFA products after a 20-day start-up period were propionate (Exp1: 65 ± 5%; Exp2: 61 ± 10% of the total COD_VFA_) and acetate (Exp1: 29 ± 5%; Exp2: 35 ± 11% of the total COD_VFA_) (Table 1, Figure 1c and 1d, and Supplementary Figure S1), with only minor contributions of (iso-)butyrate and iso-valerate (<4% of the total VFA) (Supplementary Figure S3). Methane production rates during Exp2 were on average 290 ± 107 mg COD_CH4_ L^−1^ d^−1^ between days 20 and 60, and increased to 900 ± 210 mg COD_CH4_ L^−1^ d^−1^ from Day 70 (Table 1 and Supplementary Figure S2). The CH_4_ concentration in the biogas was on average 58 ± 6 vol%, CO_2_ concentration was 33 ± 4 vol%, and H_2_ was rarely detected (<0.02 vol%).

#### Thermophilic – 12 day SRT reactor (T12)

T12 showed the most stable performance of the three experimental reactors, likely as a result of the longer SRT. Residual cellulose concentrations (pCOD) were on average 1.6 ± 0.5 g pCOD L^−1^ during Exp1 and 2.0 ± 0.5 g pCOD L^−1^ during Exp2 (Table 1 and Figure 1a). The average cellulose hydrolysis efficiency was 86 ± 4% during Exp1 and 83 ± 4% during Exp2 (Supplementary Figure S1), which was similar to the hydrolysis efficiency observed in the parent reactor (M12). Accumulation of VFAs was also observed relative to the parent reactor (M12), and increased to 4.8 ± 0.1 and 5.8 ± 0.4 g COD_VFA_ L^−1^ during Exp1 and Exp2, respectively (Figure 1b). The main VFAs produced were acetate (Exp1: 83 ± 2%; Exp2: 80 ± 3% of the total COD_VFA_), propionate (Exp1: 12 ± 2%; Exp2: 13 ± 2% of the total COD_VFA_) (Table 1, Figure 1c and 1d, and Supplementary Figure S1) and butyrate (Exp1: 3 ± 0.5%; Exp2: 5 ± 3% of the total COD_VFA_) (Supplementary Figure S3). Low concentrations of ethanol (maximum 0.25 g COD_ethanol_ L^−1^) were sporadically detected over the course of Exp1 and Exp2 (Supplementary Figure S3). Methane was produced at an average rate of 210 ± 90 mg COD_CH4_ L^−1^ d^−1^ during Exp2 (Table 1 and Supplementary Figure S2). The biogas was composed of 56 ± 3 vol% CH_4_, 37 ± 3 vol% CO_2_ and H_2_ was detected at concentrations below 0.1 vol%.

#### Thermophilic – 4 day SRT reactor (T4)

The combination of increased temperature and decreased SRT resulted in less efficient hydrolysis with peak pCOD concentrations of 14.8 and 8.8 g pCOD L^−1^ during Exp1 and Exp2, respectively, after 20 days of operation (Figure S1). Over time reactor performance became more stable and the average pCOD concentration was 5.6 ± 0.9 g COD L^−1^ between Days 30 and 90 of Exp2 (Figure 1a), corresponding to an average cellulose degradation efficiency of 54 ± 8%. VFA concentrations increased to 6.3 ± 0.2 during Exp1 and 4.4 ± 0.6 g COD_VFA_ L^−1^ during Exp2. The main VFA produced during both experiments was acetate (Exp1: 67 ± 4%; Exp2: 86 ± 5% of the total COD_VFA_), with low amounts of propionate (Exp1: 19 ± 4%; Exp2: 11 ± 3% of the total COD_VFA_) (Table 1, Figure 1c and 1d, and Supplementary Figure S1), butyrate (Exp1: 8 ± 5%; Exp2: <4% of the total COD_VFA_) and ethanol (maximum 0.55 g COD_ethanol_ L^−1^) also produced (Supplementary Figure S3). Although the overall methane production rate in T4 (605 ± 190 mg COD_CH4_ L^−1^ d^−1^) was higher than in the mesophilic reactors (M4 and M12) and T12 (Table 1 and Supplementary Figure S2), the relative percentage of feed COD converted to methane was lowest in T4 (24 ± 5%). The produced biogas consisted of 63 ± 5 vol% CH_4_ and 29 ± 7 vol% CO_2_, and H_2_ was detected at concentrations below 0.1 vol%.

### Microbial community composition and dynamics

**Figure 2:**
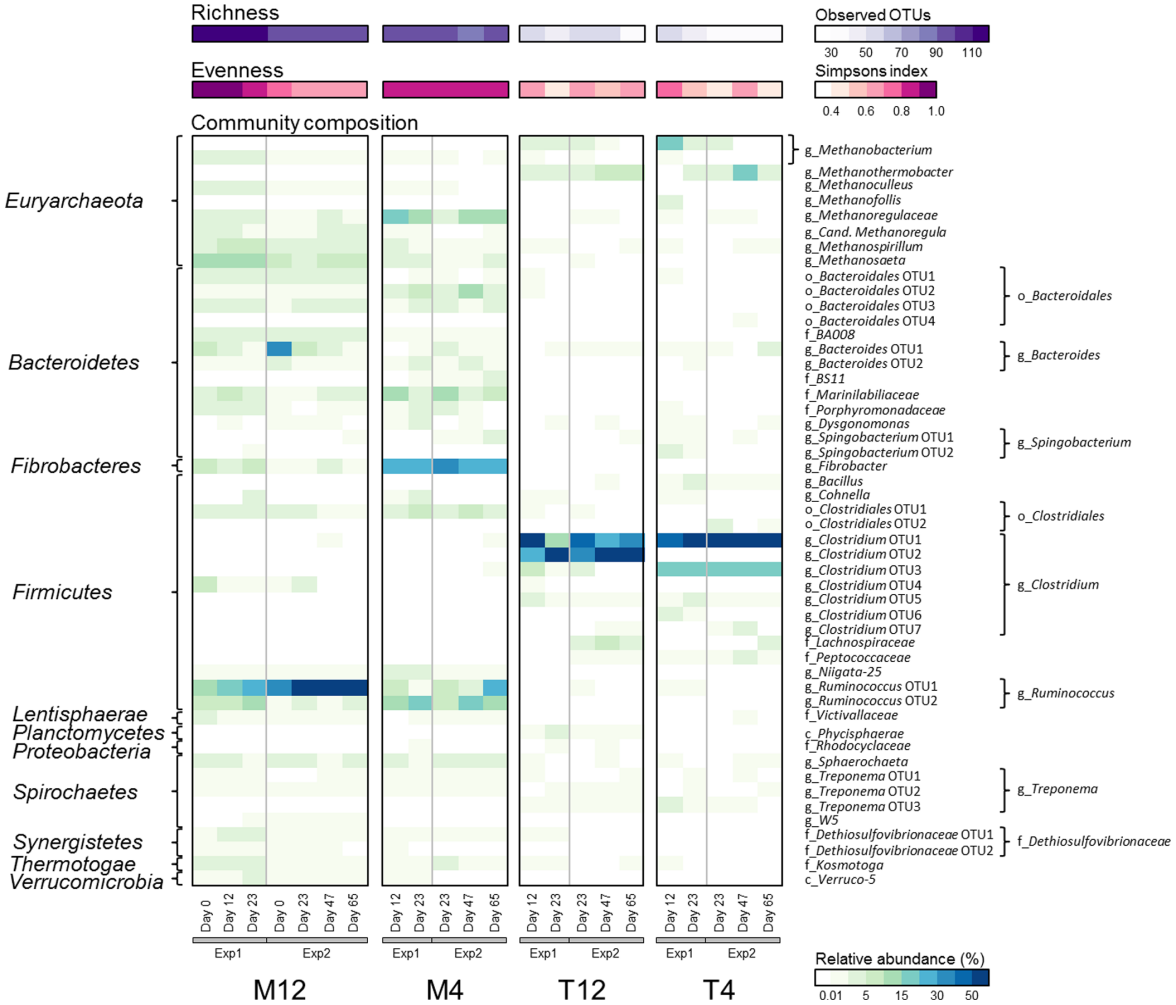
Microbial community richness (observed operational taxonomic units (OTUs)), evenness (Simpson index) and composition (relative abundance) of the parent (M12) and experimental reactors (M4, T12 and T4). The relative abundances of populations detected at >1% in at least one of the samples are shown. The Day 0 sample from reactor M12 was the inoculum for the experimental reactors (both experiments). Each row in the heatmap represents an OTU and taxonomic classifications based on the 16S rRNA gene are shown at the phylum level (left hand side) and lowest level of taxonomic assignment (c: class, o: order, f: family, and g: genus; right hand side).

The microbial community composition of each reactor was characterized over time using 16S rRNA gene amplicon sequencing in order to monitor the influence of temperature and SRT on the community structure and dynamics (Exp1: Days 12 and 23; Exp2: Days 23, 47 and 65). The parent reactor (M12) was dominated by populations belonging to the bacterial genera *Ruminococcus*, *Clostridium*, *Bacteroides* and the archaeal genus *Methanosaeta* (Figure 2). During both experiments, changes in operating conditions led to a decrease in richness for all experimental reactors from 102 ± 12 observed OTUs in M12 to 93 ± 6 in M4 (*P* > 0.05), 48 ± 7 in T12 and 42 ± 11 in T4 (*P* < 0.05), and a decrease in evenness for the thermophilic reactors from 0.78 ± 0.13 in M12 to 0.58 ± 0.10 in T12 and 0.55 ± 0.12 in T4 (*P* < 0.05) (Figure 2 and Supplementary Table S1). The average community evenness in M4 during both experiments showed a slight increase to 0.86 ± 0.02 (*P* > 0.05) (Figure 2 and Supplementary Table S1). While the microbial community composition differed among reactors, the communities that developed in each reactor were highly similar in Exp1 and Exp2 (*P* < 0.05), demonstrating that operating conditions had a strong selective and reproducible effect on the microbial community richness, evenness, structure and function (Figure 2).

Under mesophilic conditions, decreasing the SRT from 12 (M12) to 4 days (M4) resulted in relative abundance shifts of some of the dominant populations. During both experiments, M4 was dominated by several members of the genera *Alkaliflexus*, *Fibrobacter* and *Ruminococcus* (Figure 2). *Fibrobacter* increased in abundance to 20–37% in M4 (range in Exp1 and Exp2) compared to a maximum of 8% in M12, while the total abundance of all *Ruminococcus* populations decreased from 44 ± 18% in M12 to 21 ± 6% in M4. The most abundant *Ruminococcus* population (OTU2) in M4 also differed (<97% nucleotide identity at the 16S rRNA gene level) from the dominant *Ruminococcus* (OTU1) in M12. There was also a shift in the dominant methanogen from an acetoclastic *Methanosaeta* (9 ± 4% in M12) to a hydrogenotrophic member of the family *Methanoregulaceae* (13 ± 5% in M4) (Figure 2).

During both experiments, the community profiles in the thermophilic reactors (T12 and T4) differed significantly from M12 (*P* < 0.05), and the reactors were largely dominated by members of the bacterial genus *Clostridium* (87 ± 3% in T12, 83 ± 9% in T4), and archaeal genera *Methanothermobacter* (4 ± 2% in T12, 6 ± 2% and a peak measurement of 20% in T4) and *Methanobacterium* (2 ± 1% in T12, 5 ± 2% and a peak measurement of 18% in T4) (Figure 2). *Clostridium* OTU1 was abundant in both thermophilic reactors, while the difference in SRT caused *Clostridium* OTU2 to become abundant in T12 and *Clostridium* OTU3 in T4. OTU1 was most closely related to *C. stercorarium* (97% similarity), while OTU2 and OTU3 were most closely related to *C. clariflavum* (98% and 99% similarity, respectively).

**Figure 3:**
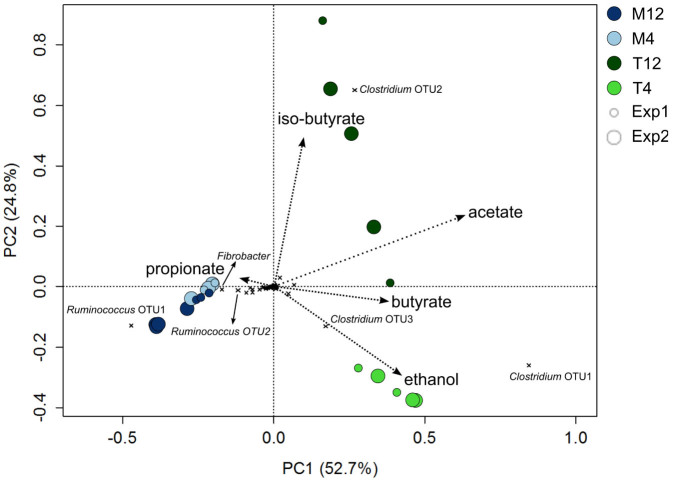
Principle component analysis showing the microbial community composition of the parent (M12) and experimental reactors (M4, T12 and T4) over time during both experiments and correlated with reactor performance parameters. Each circle represents a sample from one of the reactors, color coded based on the operating temperature (mesophilic: blue; thermophilic: green), color shading based on the SRT (4 day: light; 12 day: dark), and circle size representing the experiment (Exp1: small; Exp2: large). The microbial populations contributing most to the variability between samples are identified on the graph. Correlations with performance parameters are indicated by the arrows and were significant for acetate, (iso-)butyrate and ethanol (*P* < 0.05).

The microbial communities in the experimental reactors rapidly shifted away from the inoculum and stayed equally dissimilar to the inoculum over time (Supplementary Figure S4). The communities showed distinct clustering according to the imposed set of operating conditions, explaining 81% of the variability between samples in a constrained principle component analysis of all samples from both experiments (*P* = 0.001) (Figure 3 and Supplementary Figure S5a). Temperature had a significant influence on the community composition (*P* = 0.001) and accounted for 50% of the variability between all samples (Supplementary Figure S5b). The communities in the mesophilic reactors (M12 and M4) grouped together and were clearly distinct from the thermophilic reactor communities (T12 and T4) (Figure 3). After eliminating the influence of temperature, there was a significant influence of SRT (*P* = 0.001), explaining 53% of the remaining variability between M12 and M4, and 68% of the remaining variability between T12 and T4 (Supplementary Figure S6). Only 2% of the variability between samples could be explained by the different experiments (*P* > 0.5), indicating that the influence of temperature and SRT on the community composition was highly reproducible.

The microbial community composition was also significantly correlated with product formation in the reactors (*P* < 0.05); particularly with the VFA profiles (Figure 3). Three OTUs in the thermophilic reactors (T4 and T12) belonging to the genus *Clostridium* showed a correlation with increased production and accumulation of acetate. The most abundant *Clostridium* in T12 (OTU2) was also correlated with higher levels of iso-butyrate, while the two most abundant *Clostridium* OTUs in T4 (OTU1 and OTU3) were correlated with higher concentrations of butyrate and ethanol (Figure 3). When the PCA was constrained by SRT, the communities in M4, and more specifically individual OTUs belonging to the genera *Fibrobacter*, *Ruminococcus* (OTU2) and *Bacteroidales* were correlated with increased propionate production (Supplementary Figure S5c).

## Discussion

In this study, reactors with varying SRT and temperature were operated using inoculum from a stably performing methanogenic digester to develop a strategy for restricting methanogenesis while maintaining efficient hydrolysis and fermentation. Using these operational parameters, we aimed to selectively drive mixed microbial communities towards increased accumulation of specific intermediate volatile fatty acids.

Reducing the SRT from 12 to 4 days at 37°C (M4) decreased the cellulose utilization by ~13% of the feed COD relative to the parent reactor (M12) (Supplementary Figure S1), likely due to the lower contact time between the cellulosic substrate and hydrolytic bacteria, and to biomass washout. The shorter SRT led to accumulation of VFAs, primarily propionate (75%) and a smaller amount of acetate (25%) (Supplementary Figure S1), consistent with prior findings on overloading of ADs at reduced SRT^25^. Although the richness did not decrease significantly between M12 and M4, there were changes in the microbial community composition. Propionate accumulation in M4 was correlated with an increased abundance of a member of the genus *Alkaliflexus*, known to be capable of propionate production^6^,^26^,^27^. Washout of syntrophic propionate oxidizers at low SRT could also potentially explain propionate accumulation, and populations within this functional guild were not detected in both M12 and M4. There was also a shift in the dominant hydrolytic populations from *Ruminococcus* OTU1 to a member of the genus *Fibrobacter* in M4. *Ruminococcus* is known to have a competitive advantage under cellulose- and cellobiose-limited conditions^28^, which may explain its higher abundance when residual cellulose concentrations were low in M12. At higher loading rates, resulting from a shorter SRT in M4, *Fibrobacter* was able to outcompete *Ruminococcus*, which may be due to their difference in cellulose attachment strategies and high cellulose hydrolysis efficiency of *Fibrobacter*^29^,^30^. Our results contradict prior studies showing more rapid attachment of some *Ruminococcus* populations to cellulose compared to *Fibrobacter*, which suggest *Ruminococcus* would have a competitive advantage at lower contact times resulting from the shorter SRT in M4^31^. These conflicting results again show the complexity of the interactions between cellulolytic populations which can be influenced by multiple factors^30^,^31^.

In M4, methane production still occurred during VFA accumulation, although yields decreased by 54% (based on hydrolyzed COD) compared to M12 (Supplementary Figure S1). This lower amount of methane was produced by a population belonging to the family *Methanoregulaceae* through hydrogenotrophic methanogenesis from formate and/or H_2_/CO_2_ derived from fermentation. Limited conversion of acetate to H_2_/CO_2_ by syntrophic acetate oxidizers (SAO), washout of the slow-growing acetoclastic *Methanosaeta* at reduced SRT, and partial inhibition of the dominant hydrogenotrophic methanogens at high VFA concentrations are possible explanations for the observed lower methane production in M4. Shortening the SRT to less than 4 days would likely further increase selective pressure against methanogenesis, however this may also result in less stable performance, reduced substrate utilization and potentially lower VFA yields. Experiments and model-based predictions are potential additional strategies to determine the optimum SRT to maximize cellulose hydrolysis and VFA accumulation with minimal methane production.

Cellulose hydrolysis at 55°C and a 12 day SRT (T12) was highly efficient, and similar to the level measured in the parent reactor (M12) (Supplementary Figure S1). The hydrolysis efficiency was expected to be higher at increased temperature^14^, however this was not observed and may be due to the already high efficiency of the parent reactor compared to other studies^9^,^14^. Another possible explanation is the lower diversity of the inoculum and the microbial communities established in these reactors compared to full-scale mesophilic and thermophilic ADs (Supplementary Table S1). Substrate complexity also has a large influence on the community composition^15^,^20^ with a lower number of potential substrates typically leading to low diversity communities that are more susceptible to changes in operating conditions^4^. The higher sensitivity of these low diversity communities to changes in operating conditions, biomass washout at reduced SRT and growth suppression at excess carbon may have caused the decrease in hydrolysis efficiency observed under thermophilic conditions and at a 4 day SRT (T4).

Increasing the temperature led to a significant decrease in microbial community richness and evenness, indicating that only a limited number of populations in the mesophilic inoculum were capable of responding to temperature increase. There was a substantial shift in community profile at the higher temperature in both T12 and T4, resulting in communities dominated by populations belonging to the genus *Clostridium* that were not detected in the inoculum (<0.0005%). This highlights the strong selective pressure of temperature, which allowed populations with a competitive advantage to become dominant at a higher temperature. *Clostridia* has previously been found as the dominant population in thermophilic ADs^20^,^32^,^33^, which is consistent with the thermophilic growth properties of members within this class. The dominant populations were closely related to *C. stercorarium* (OTU1) and *C. clarivlavum* (OTU2 and OTU3), which are known anaerobic thermophilic bacteria capable of hydrolyzing cellulose to produce acetate and ethanol^34^,^35^,^36^,^37^. The relative distribution of these *Clostridium* populations was influenced by the difference in SRT leading to dominance of OTU1 and OTU2 in T12, and OTU1 and OTU3 in T4, which correlated with differences in ethanol and (iso-)butyrate production. The main VFA product in both T12 and T4 was acetate, which accounted for >50% of the hydrolyzed COD in both reactors (Supplementary Figure S1). Acetate accumulation was significantly correlated with an increased abundance of the *Clostridium* populations (OTU1, OTU2 and OTU3), suggesting these populations played a large role in acetate production at high temperatures. Due to the lack of functional information, the specific mechanism for acetate accumulation in T12 and T4 could not be identified; however common pathways for acetate production that may have been stimulated at high temperatures are direct fermentation of glucose to acetate and conversion of higher chain VFAs to acetate via acetogenesis. While propionate accumulation has been observed at elevated temperatures^11^, concentrations remained relatively low in T12 and T4 compared to the acetate accumulation in these reactors and the propionate concentration in M4 (Supplementary Figure S1). This was likely due to a combination of lower propionate production and effective conversion to acetate at low partial H_2_ pressures.

Acetate consumption generally occurs through SAO or direct cleavage by acetoclastic methanogens. As acetate consuming methanogens were not detected in the thermophilic reactors, direct cleavage was likely to be very limited. Recent studies have identified syntrophic acetate oxidation linked to hydrogenotrophic methanogenesis in thermophilic AD^14^,^38^, however the observed high acetate concentrations in T12 and T4 suggest that populations performing SAO were not present in the reactors or only present at very low abundance. H_2_-consuming methanogens belonging to the genera *Methanothermobacter* and *Methanobacterium* were the dominant methanogens in T12 and T4. Although methane production was lower under thermophilic conditions, 30%–40% of hydrolyzed COD in these reactors was still being converted to methane through hydrogenotrophic methanogenesis. Our findings are consistent with previous research showing that consumption of H_2_ by *Methanothermobacter* and *Methanobacterium* during glucose fermentation at high temperatures (70°C) assists in selective and stable production of acetate^13^.

Replication of the experiment clearly demonstrated high reproducibility in terms of both changes in VFA production profiles and microbial community composition under each set of operating conditions. Previously, both niche and neutral ecological theories have been applied to describe the factors driving changes in microbial community function and structure^18^,^22^,^23^. The reproducible results from this study highlight the importance of niche differentiation allowing more competitive populations to become dominant when conditions change, and underline the predominant role of deterministic processes such as operating conditions, substrate availability and microbial interactions in anaerobic digestion^16^.

In this study, we demonstrate that VFA accumulation can be achieved at relatively high concentrations with reduced levels of methane production, while maintaining a stable microbial community. This outcome demonstrates the potential for novel carboxylate processes to produce both high value products and renewable energy in a single reactor. However, it should be emphasized that the use of a different substrate would likely result in a different microbial community and product profile. The process conditions should therefore be optimized for biotechnological applications depending on the substrate-product combination required.

In terms of controlling product formation, increasing the temperature and shortening the SRT both resulted in VFA accumulation, however the type of VFA produced was predominantly driven by temperature. Additional methods could be explored to further enhance VFA production, such as lowering the pH to increase product yield and specificity, and extracting products to eliminate thermodynamic constraints and release toxicity pressures on hydrolysis and fermentation^8^,^39^. Furthermore, meta-omic analyses and substrate labelling methods would allow us to identify the mechanisms responsible for the accumulation of specific VFAs under the different operating conditions^40^, which in turn will lead to further process optimization. The outcomes from this study demonstrate that microbial communities performing AD can be driven towards enhanced production of specific high value VFAs in a controlled and replicated manner by using selective operating conditions.

## Methods

### Reactor set-up and operation

A parent anaerobic digester (continuously stirred tank reactor (CSTR); 4 L working volume) was operated for 250 days and showed stable conversion of alpha cellulose to methane (Supplementary Figure S7). This parent reactor, designated as M12, was used to seed three smaller experimental reactors (CSTR; 2 L working volume), further referred to as M4, T12 and T4. The waste stream from the parent reactor was collected over a three week period and used as inoculum. The anaerobic digesters were run as mixed semi-continuous reactors at four sets of operating conditions: Parent M12 – mesophilic (37°C) at a 12 day SRT, M4 – mesophilic (37°C) at a 4 day SRT, T12 – thermophilic (55°C) at a 12 day SRT, and T4 – thermophilic (55°C) at a 4 day SRT (Table 2). In order to avoid acidification of the reactors and eliminate the influence of pH, 1 M NaOH was added regularly to maintain a pH of 7. Two independent experiments were performed at each set of experimental conditions to assess the reproducibility of reactor performance and to determine the effect these changes have on the microbial community composition. Experiment 1 (Exp1) was a short-term experiment run for 34 days, and Experiment 2 (Exp2) was run for 90 days to monitor performance stability and community dynamics over time.

Alpha cellulose (Sigma Aldrich, NSW Australia), which is the insoluble and highly polymerized fraction in cotton^41^, was added as a model substrate because it is a more natural substrate than carboxymethyl cellulose (CMC). The sterile medium consisted of 3 g L^−1^ Na_2_HPO_4_, 1 g L^−1^ NH_4_Cl, 0.5 g L^−1^ NaCl, 0.2465 g L^−1^ MgSO_4_.7H_2_O, 1.5 g L^−1^ KH_2_PO_4_, 14.7 mg L^−1^ CaCl_2_, 2.6 g L^−1^ NaHCO_3_, 0.5 g L^−1^ C_3_H_7_NO_2_S, 0.25 g L^−1^ Na_2_S.9H_2_O, and 1 mL of trace solution containing 1.5 g L^−1^ FeSO_4_.7H_2_O, 0.15 g L^−1^ H_3_BO_3_, 0.03g L^−1^ CuSO_4_.5H_2_O, 0.18 g L^−1^ KI, 0.12 g L^−1^ MnCl_2_.4H_2_O, 0.06 g L^−1^ Na_2_Mo_4_.2H_2_O, 0.12 g L^−1^ ZnSO_4_.7H_2_O, 0.15 g L^−1^ CoCl_2_.6H_2_O, 10 g L^−1^ EDTA and 23 mg L^−1^ NiCl_2_.6H_2_O^16^. During preparation, the medium was sparged with nitrogen gas followed by autoclaving at 121°C for 60 min to ensure the medium was anoxic and sterile. Hydrogen chloride (37 vol%) was added to adjust the pH of the medium to ~7.2. The reactors were fed semi-continuously with alpha cellulose (12 g cellulose L^−1^_medium_) at six-hourly intervals, during which approximately 125 mL (M4 and T4), 80 mL (M12) or 40 mL (T12) of feed was pumped in the systems and an equal amount of reactor sludge was wasted simultaneously using multi-head peristaltic pumps (John Morris Scientific, QLD Australia). This resulted in an organic loading rate (OLR) of 3 g alpha cellulose L^−1^_reactor volume_ d^−1^ for M4 and T4, and 1 g alpha cellulose L^−1^_reactor volume_ d^−1^ for M12 and T12.

### Chemical analyses

Samples were collected from each reactor three times per week and analyzed for total chemical oxygen demand (tCOD), soluble COD (sCOD) and VFA concentrations. COD analyses were performed using Merck Spectroquant® COD cell tests and a SQ118 Photometer (Merck, Germany). tCOD was measured using concentration range 500–10,000 mg L^−1^ cells on unfiltered samples and sCOD was measured using concentration range 25–1,500 mg L^−1^ cells on filtered samples after dilution. VFA concentrations were measured using a gas chromatograph (Agilent Technologies, Model 7890A, USA) with a flame ionization detector (FID) and a polar capillary column (DB-FFAP) on filtered samples after dilution and addition of an internal standard (1000 ppm stock of six VFAs) and 1% formic acid. The biogas production rate was calculated daily during the second trial based on continuous cumulative gas measurements using tipping bucket gas meters. Gas composition (CH_4_, CO_2_, H_2_) was determined using a GC with a terminal conductivity detector (PerkinElmer, Model 1022, USA)^14^.

### DNA extraction and 16S rRNA gene amplicon sequencing

Samples for DNA extraction were taken twice per week, snap-frozen in liquid nitrogen and stored at −80°C until processing. DNA extractions were performed using FastDNA Spin Kits for Soil (MP Biomedicals, NSW Australia), according to the manufacturer's instructions. DNA concentrations were measured using Quant-iT dsDNA BR Assay kits with a Qubit fluorometer (Life Technologies, VIC Australia).

Genomic DNA was extracted from samples taken at days 0, 12 and 23 during Exp1 and at days 0, 23, 47 and 65 during Exp2. The V6–V8 regions of the bacterial and archaeal 16S rRNA gene was amplified and sequenced at the Australian Centre for Ecogenomics using the Roche 454 GS-FLX Titanium platform, as described previously^16^. Sequences were submitted to the NCBI Short Read Archive under the following accession numbers: SRR1531159 (Exp1) and SRR1531160 (Exp2).

Amplicon sequences were filtered based on quality, trimmed to 250 bp and dereplicated using QIIME. Chimeric sequences were removed with UCHIME and homopolymer errors were corrected using Acacia. CD-Hit OTU was used to cluster sequences at 97% operational taxonomic unit (OTU) identity and BLASTn was used to assign a Greengenes taxonomy to cluster representatives. The 97% OTU datasets were repeatedly subsampled to calculate the number of observed OTUs at equal number of sequences (richness) and Simpsons Diversity Index (evenness). The datasets were normalized to 2100 sequences to eliminate bias from unequal sampling depth. A normalized OTU table was generated with a list of all OTUs, their taxonomy and relative abundance within each sample. 16S rRNA gene sequences of OTUs of interest were aligned against the Greengenes database and sequences were inserted into the reference tree using maximum parsimony in ARB to determine closely related representative sequences.

### Statistical analyses

Statistical analyses were performed using R Studio (version 2.15.0) and the R CRAN packages: vegan and RColorBrewer. Differences in microbial community composition were explored and visualized using complete hierarchal clustering, heatmaps and principle component analysis (PCA). The Euclidian distance was calculated to determine the similarity between the parent and experimental reactor communities. Reactor performance parameters, OTU abundances, richness and evenness were compared between experiments and between reactors using Tukey Honestly Significant Differences Tests (TukeyHSD). Correlations between microbial community composition and reactor performance parameters were calculated using the environmental parameter fitting function in R.

## Author Contributions

I.V. carried out the experiment, analyzed the data and wrote the paper. K.R. helped conceptualize the project and reviewed the paper. P.D.J. and G.W.T. were involved in the design of the experiment, and helped interpret the data and write the paper.

## Supplementary Material

Supplementary Information Supplementary info

## Figures and Tables

**Table 1:** Overview of the residual cellulose and conversion products shown as concentrations and percentages of the feed COD in the parent (M12) and experimental reactors (M4, T12, T4) during Exp2 after 65 days of operation

Reactor	Residual cellulose		Soluble compounds		VFAs total		Acetate		Propionate		Methane	
	g COD_pCOD_ L^−1^	%COD_feed_	g COD_sCOD_ L^−1^	%COD_feed_	g COD_VFA_ L^−1^	%COD_feed_	mg COD_acetate_ L^−1^	%COD_feed_	g COD_propionate_ L^−1^	%COD_feed_	g COD_CH4_ L^−1^ d^−1^	%COD_feed_
Parent M12	2.5 ± 0.1	20 ± 1	0.5 ± 0.04	4 ± 0.3	0.06 ± 0.02	0.5 ± 0.1	0.05 ± 0.01	0.5 ± 0.1	<0.01	<0.1	0.7 ± 0.1	72 ± 11
M4	4.0 ± 7.5	33 ± 6	5.0 ± 0.6	42 ± 5	4.9 ± 0.6	41 ± 5	1.2 ± 0.5	10 ± 4	3.4 ± 0.3	28 ± 3	0.8 ± 0.2	29 ± 7
T12	2.2 ± 0.4	18 ± 3	5.7 ± 0.3	48 ± 2	6.0 ± 0.3	50 ± 3	4.9 ± 0.2	41 ± 2	0.8 ± 0.06	7 ± 0.5	0.2 ± 0.08	26 ± 11
T4	5.1 ± 0.7	43 ± 6	4.4 ± 0.2	37 ± 2	4.2 ± 0.3	35 ± 3	3.7 ± 0.2	31 ± 2	0.4 ± 0.07	3 ± 0.6	0.7 ± 0.2	24 ± 5

**Table 2:** Design operating conditions (temperature, SRT and OLR) for the parent (M12) and experimental reactors (M4, T12, T4)

Reactor	Temperature (°C)	SRT (days)	OLR (g COD L^−1^ d^−1^)
Parent M12	37	12	1
M4	37	4	3
T12	55	12	1
T4	55	4	3
