# Supplementary material for: Temperature and solids retention time control microbial population dynamics and volatile fatty acid production in replicated anaerobic digesters
Source: Sci Rep. 2015 Feb 16;5:8496. doi: 10.1038/srep08496 (PMC4329568; doi:10.1038/srep08496)
Supplement: Supplementary Information — Supplementary info [file srep08496-s1.pdf]

Temperature and solids retention time control microbial population dynamics and volatile fatty acid production in replicated anaerobic digesters

Inka Vanwonterghem[1,2], Paul D Jensen[1], Korneel Rabaey[1,3] and Gene W Tyson[1,2*]

[1]Advanced Water Management Centre (AWMC), The University of Queensland, St Lucia, QLD 4072, Australia; [2]Australian Centre for Ecogenomics (ACE), School of Chemistry and Molecular Biosciences, The University of Queensland, St Lucia, QLD 4072, Australia; [3]Laboratory for Microbial Ecology and Technology (LabMET), Ghent University, Coupure Links 653, 9000 Ghent, Belgium

Submitted for publication in Scientific Reports

***Corresponding author:** A/Prof. Gene W. Tyson. Mailing address: Australian Centre for Ecogenomics (ACE), School of Chemistry and Molecular Biosciences, The University of Queensland, St Lucia, QLD 4072, Australia. Phone: +617 3365 3829 Fax: +617 336 54511 Email: g.tyson@uq.edu.au

SUPPLEMENTARY INFORMATION

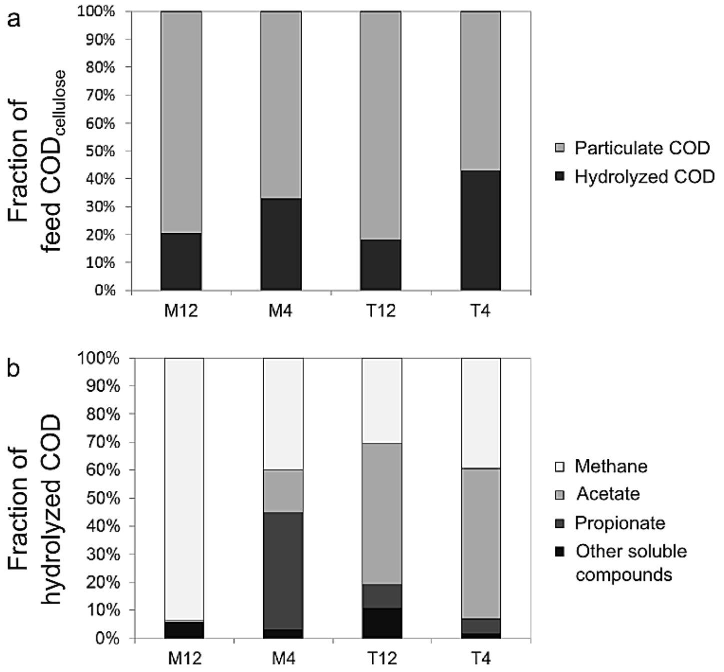

Supplementary Figure S1. Overview of the cellulose conversion in the parent (M12) and experimental reactors (M4, T12, T4) during Exp2 after 65 days of operation. a) Fraction of the feed COD present in the reactors as residual particulates and hydrolyzed compounds. b) Distribution of end products as a fraction of hydrolyzed COD.

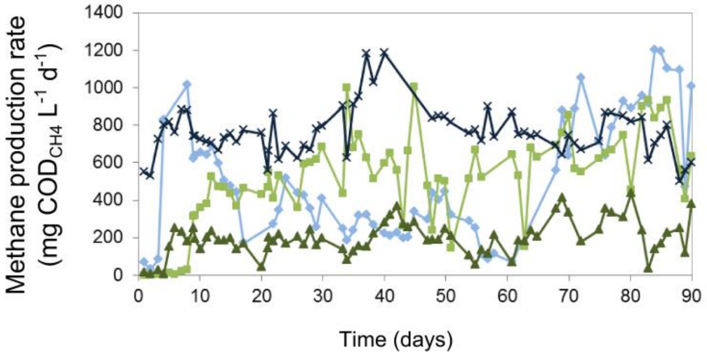

Supplementary Figure S2. Methane production rate over time for the parent (M12: × dark blue) and experimental reactors (M4: ◊ light blue; T12: Δ dark green; T4: □ light green) during Exp2. Additional measurements between days 40 and 50 were missing for M12 due to a problem with the gas meter.

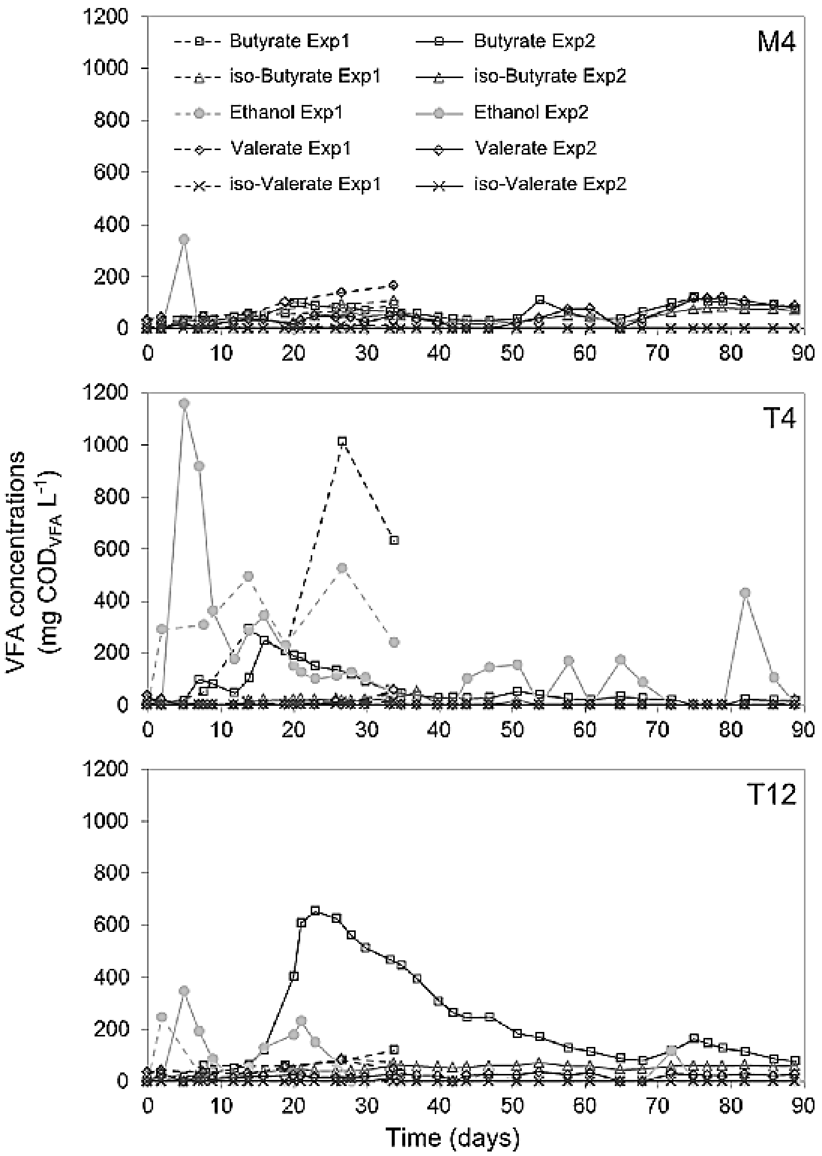

Supplementary Figure S3. VFA concentrations over time for the experimental reactors (M4, T4, T12) during both experiments (Exp1: dashed line; Exp2: full line). Concentrations are shown for butyrate (□), iso-butyrate (Δ), ethanol (○), valerate (◊) and iso-valerate (×). VFA profiles for M12 are not shown and were < 50 mg COD_{VFA} L^{-1}.

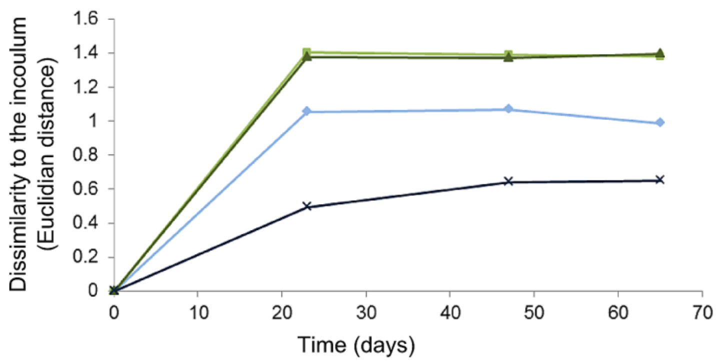

Supplementary Figure S4. Euclidian distance (dissimilarity) of the microbial communities in the parent (M12: × dark blue) and experimental reactors (M4: ◊ light blue; T12: Δ dark green; T4: □ light green) to the inoculum over time during Exp2.

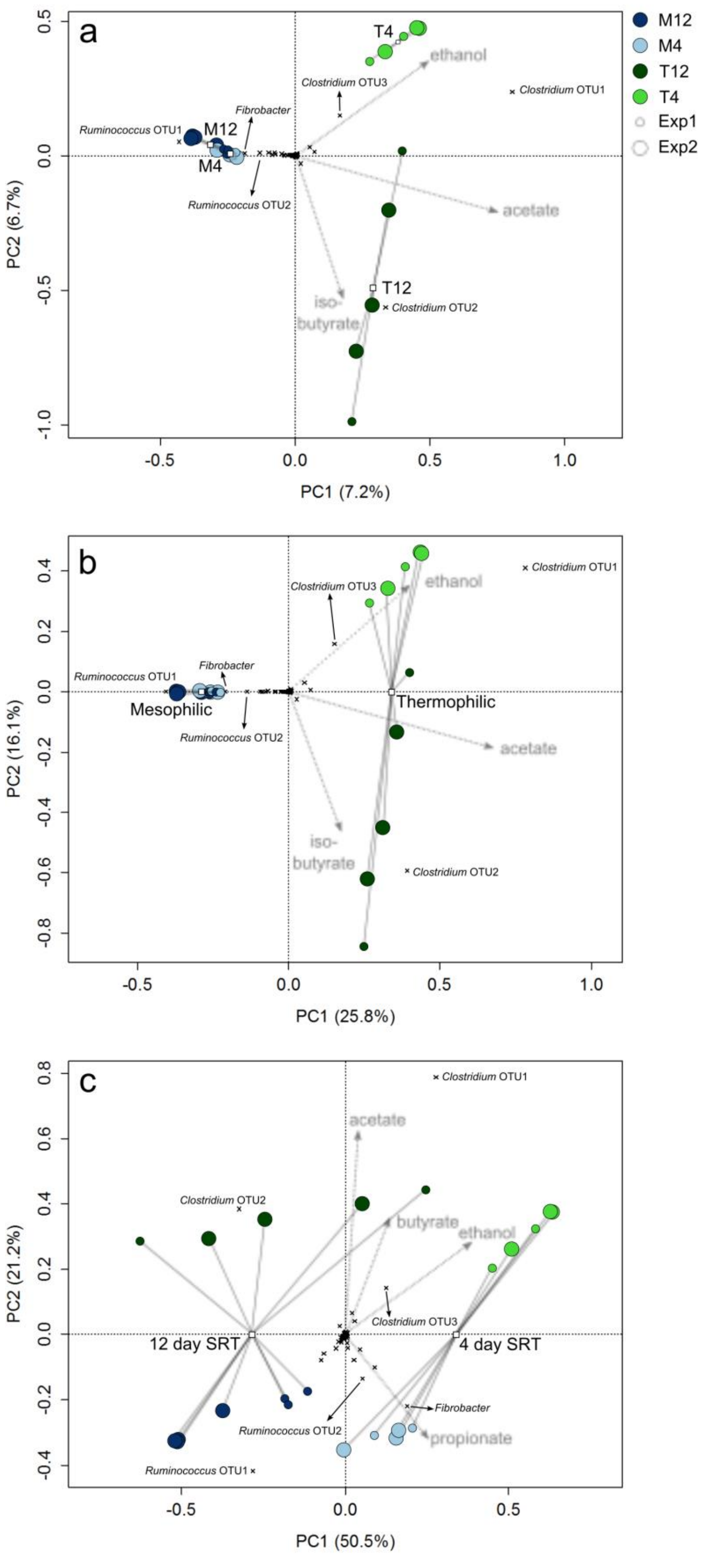
a
M12
M4
T12
T4
Exp1
Exp2
PC1 (7.2%)
PC2 (6.7%)
Clostridium OTU1
Clostridium OTU2
Clostridium OTU3
Fibrobacter
Ruminococcus OTU1
Ruminococcus OTU2
ethanol
acetate
iso-
butyrate
b
Mesophilic
Thermophilic
PC1 (25.8%)
PC2 (16.1%)
c
12 day SRT
4 day SRT
butyrate
propionate
PC1 (50.5%)
PC2 (21.2%)

Supplementary Figure S5. Principle component analysis showing the microbial community composition of the parent (M12) and experimental reactors (M4, T12, T4) over time during both experiments. The PCAs were constrained by operating parameters: a) Reactor (P = 0.001); b) Temperature (P = 0.001); c) SRT (P = 0.018). Color coding is based on the operating temperatures (mesophilic: blue; thermophilic: green), color shading is based on the SRT (4 day: light; 12 day: dark), and circle size represents the experiment (Exp1: small; Exp2: large). The microbial populations contributing most to the variability between samples are identified on the graph and significant correlations with performance parameters are indicated by the arrows ($P < 0.05$).

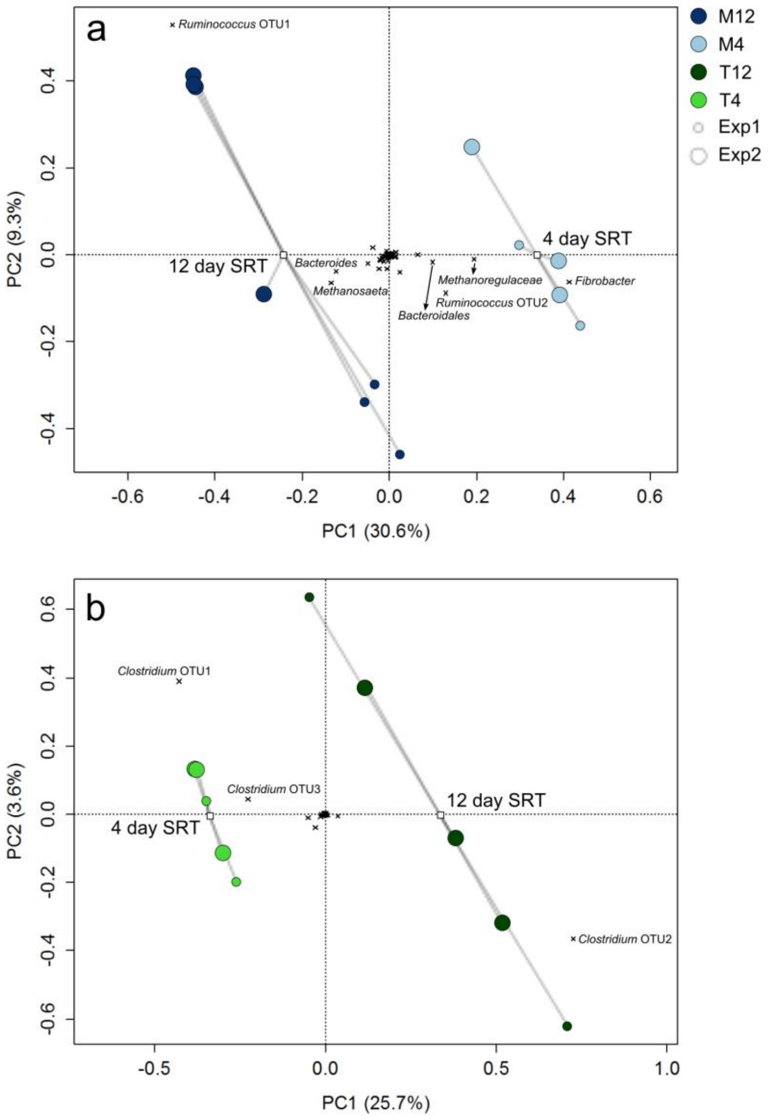

Supplementary Figure S6. Principle component analysis showing the microbial community composition of the parent (M12) and experimental reactors (M4, T12, T4) over time during both experiments. The PCAs were constrained by SRT after separation based on temperature: a) Mesophilic; b) Thermophilic. Color coding is based on the operating temperatures (mesophilic: blue; thermophilic: green), color shading is based on the SRT (4 day: light; 12 day: dark), and circle size represents the experiment (Exp1: small; Exp2: large). The microbial populations contributing most to the variability between samples are identified on the graph.

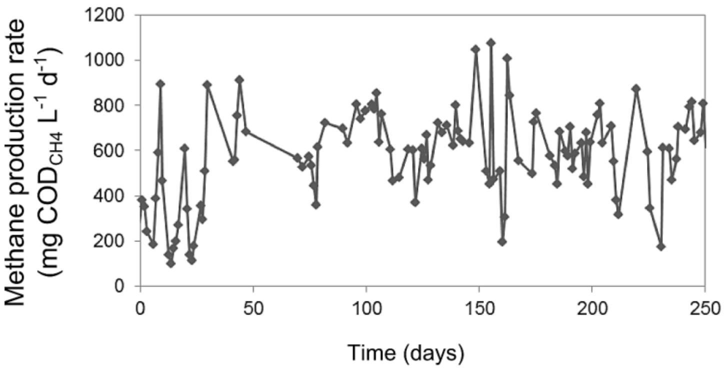

Supplementary Figure S7. Methane production rate over time for the parent (M12) prior to inoculation, showing stable performance.

Supplementary Table S1. Comparison of the richness (observed OTUs) and evenness (Simpson diversity) of the parent (M12) and experimental reactors (M4, T12, T4; averaged for the two experiments) to full-scale AD systems and a rumen sample.

**Source**	**Description**	**Richness**	**Evenness**
Parent M12	Parent reactor (37°C, 12 day HRT)	102 ± 12	0.78 ± 0.13
M4	Experiment reactor (37°C, 4 day HRT)	93 ± 6	0.86 ± 0.02
T12	Experiment reactor (55°C, 12 day HRT)	48 ± 7	0.58 ± 0.10
T4	Experiment reactor (55°C, 4 day HRT)	42 ± 11	0.55 ± 0.12
Mes AD1	Mesophilic digester (35°C, 30 day HRT) - waste activated sludge	172	0.87
Mes AD2	Mesophilic digester (35°C, 30 day HRT) - mixed activated sludge and primary sludge	183	0.88
Therm AD	Thermophilic digester (55°C, 2 day HRT) - first stage in TPAD - beef slaughterhouse solid waste	74	0.83
UASB	Anaerobic granules from an Upflow Anaerobic Sludge Blanket bioreactor (UASB) - acidified brewery wastewater	39	0.44
Lagoon	Anaerobic lagoon – pig manure	81	0.58
Rumen	Rumen fluid collected from a cattle paunch	165	0.94
